# Supplementary material for: Purely elastic turbulence in pressure-driven channel flows
Source: Proc Natl Acad Sci U S A. 2024 Feb 20;121(9):e2318851121. doi: 10.1073/pnas.2318851121 (PMC10907231; doi:10.1073/pnas.2318851121)
Supplement: Supplementary file 1 — Appendix 01 (PDF) [file pnas.2318851121.sapp.pdf]

Supporting Information for

Purely elastic turbulence in pressure-driven channel flows

Martin Lellep, Moritz Linkmann and Alexander Morozov

Alexander Morozov.
E-mail: alexander.morozov@ed.ac.uk

This PDF file includes:

Figs. S1 to S3
Table S1
Legends for Movies S1 to S3

Other supporting materials for this manuscript include the following:

Movies S1 to S3

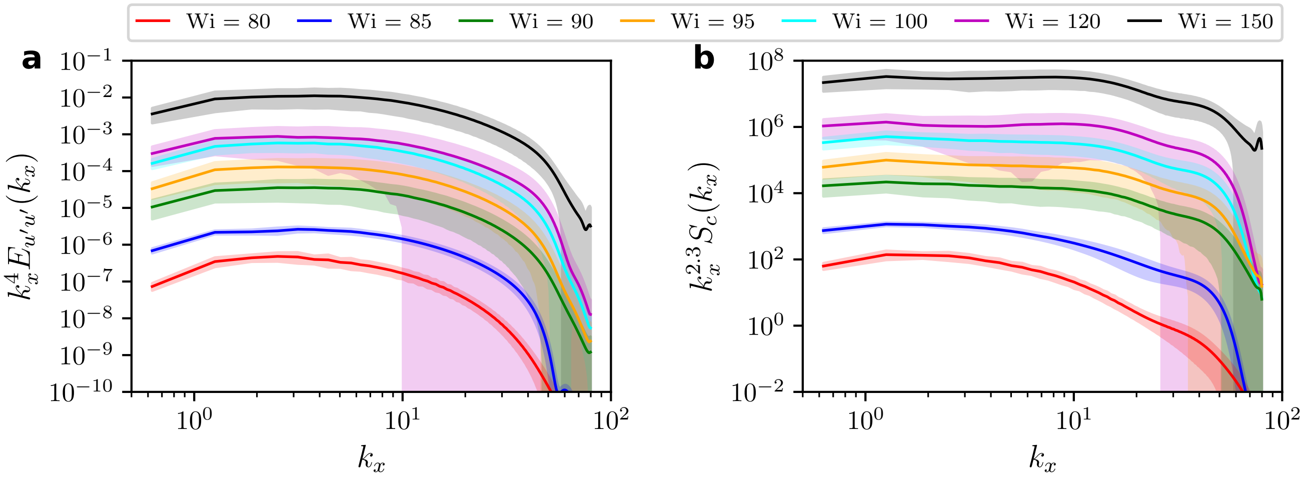

Fig. S1. Compensated spectra at different Weissenberg numbers. a, One-dimensional spectrum of the streamwise velocity component. **b**, One-dimensional spectrum of the trace of the conformation tensor. The shaded regions indicate one standard deviation. Data have been shifted vertically by the same offset in both subfigures to improve the readability of the figure.

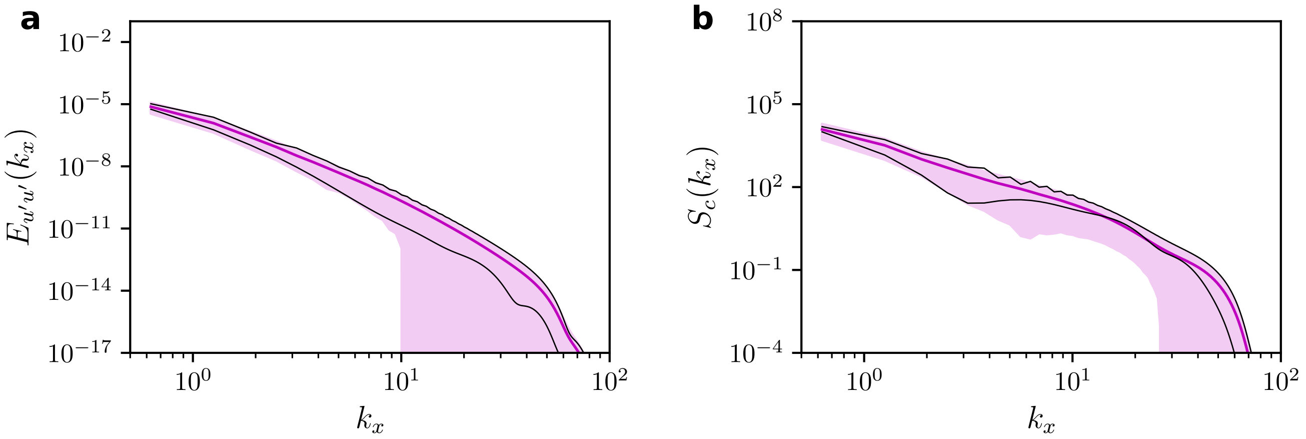

Fig. S2. Instantaneous spectra for $\mathrm{Wi} = 120$**. a**, One-dimensional spectrum of the streamwise velocity component. **b**, One-dimensional spectrum of the trace of the conformation tensor. The shaded regions indicate one standard deviation. The violet lines are ensemble-averaged spectra, while the black lines are representative instantaneous realisations.

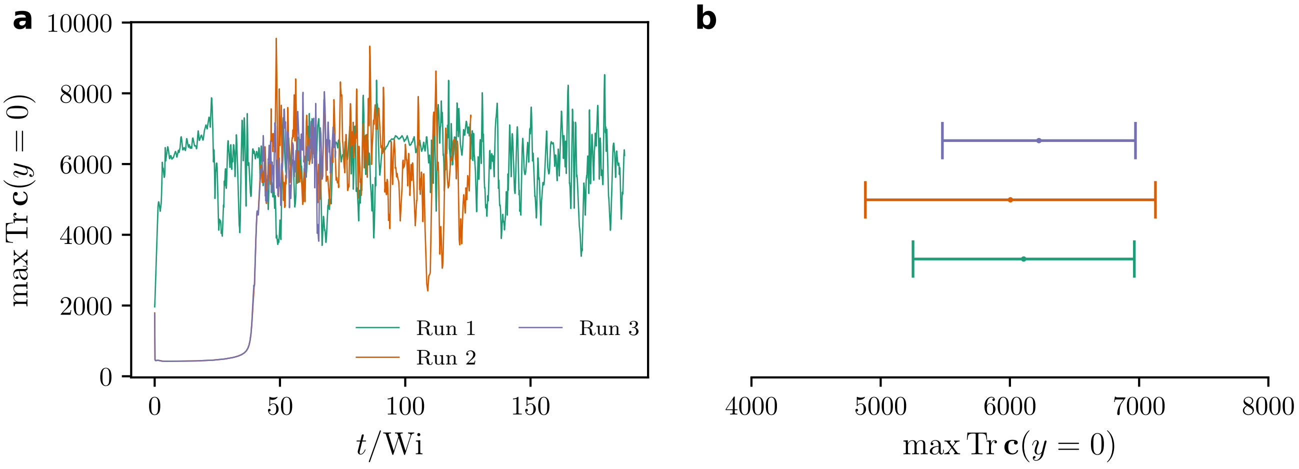

Fig. S3. Studying the influence of the initial condition and spatial resolution for $\mathrm{Wi} = 150$. a, Time evolution of the midplane polymer stretch. Run 1 employed a configuration taken from the $\mathrm{Wi} = 100$ simulation as its initial condition. The data is re-plotted from Fig.1c of the main text. Runs 2 and 3 were started from the laminar state perturbed by random noise with the amplitude $\delta = 625$ (Materials and Methods). The small-t data of Run 2 are plotted in Fig.1b. Runs 1 and 2 employed the production spatial resolution, while Run 3 employed a reduced (halfed) resolution (Materials and Methods). **b**, Statistical analysis of the data from (a) evaluated in the statistical steady state. The circles denote the averages, while the lines indicate one standard deviation. In the absence of statistically-significant or systematic discrepancies between the runs, we conclude that all three simulations have converged to the same chaotic steady state.

Table S1. Parameters and key observables used in the statistical analyses. E and $\langle \mathrm{Tr}\, \boldsymbol{c} \rangle$ are the volume- and time-averaged kinetic energy and the trace of the conformation tensor, respectively, while E_{lam} and $\mathrm{Tr}\, \boldsymbol{c}_{lam}$ are the corresponding laminar values. t_{start} (t_{stop}) is the starting (stopping) time of the interval for the calculation of statistical observables and M is the number of data samples used in the calculation of statistical observables.

Wi	E	E_{lam}	$\langle \mathrm{Tr}\, \boldsymbol{c} \rangle$	$\mathrm{Tr}\, \boldsymbol{c}_{lam}$	t_{start}/Wi	t_{stop}/Wi	M
50	1.308	1.308	1213	1213	-	-	-
75	1.357	1.357	1769	1769	-	-	-
80	1.362	1.364	1882	1874	0	52	415
85	1.368	1.370	1990	1977	60	172	955
90	1.367	1.376	2127	2079	0	60	540
95	1.373	1.382	2231	2179	0	145	1374
100	1.377	1.387	2339	2277	0	145	1459
120	1.396	1.404	2688	2656	60	134	896
150	1.406	1.423	3353	3191	22	151	1935

Movie S1. Elastic turbulence at $\mathrm{Wi} = 80$. The simulation rapidly converges to a localised turbulence structure that persists for a long time before suddenly relaminarising.

Movie S2. Elastic turbulence at $\mathrm{Wi} = 100$. The simulation is started from a two-dimensional ‘narwhal’ state, translationally-invariant along the spanwise direction, perturbed by a small amount of noise. Early time evolution shows that this state is unstable and the simulation quickly reaches a chaotic steady state.

Movie S3. Elastic turbulence at $\mathrm{Wi} = 150$. A strongly intermittent simulation exhibiting splitting and merging of localised coherent structures.
